# Assessing the Risk of Commercial Vaccines Against Pseudorabies Virus in Cats

**DOI:** 10.3389/fvets.2022.857834

**Published:** 2022-04-14

**Authors:** Lu Tu, Jingjie Zhao, Qiuyang Chen, Shan Zhang, Lin Liang, Xinming Tang, Shaohua Hou, Weifang Yang, Ruiying Liang

**Affiliations:** ^1^Institute of Animal Sciences, Chinese Academy of Agricultural Sciences, Beijing, China; ^2^Beijing General Station of Animal Husbandry, Beijing, China

**Keywords:** pseudorabies virus, attenuated vaccine, cat, epidemiology, pathogenicity

## Abstract

Pseudorabies virus (PRV) is a zoonotic agent that causes significant economic losses in animal husbandry worldwide, and *gE*-deleted vaccines play an important role in its treatment in the swine industry. However, the potential risk of attenuated PRV strains in commercial vaccines for other hosts remains unclear. Especially, cats are important companion animals for human beings. In this study, we investigated the prevalence and pathogenicity of the PRV wild strain in the cat population. We found that the occurrence of PR diseases in cats is sporadic, that the attenuated PRV strain causes slight clinical signs in cats, and that the virus is excreted 3 days post-infection. Our findings will be beneficial in furthering our understanding of the epidemiology and pathogenicity of PRV in cats and implying the great risk of RPV transmission from pigs to cats.

## Introduction

Pseudorabies virus (PRV) causes a viral disease of economic significance since it affects animal husbandry worldwide (1). The causative agent of this disease belongs to the genus *Varicellovirus* of the subfamily *Alphaherpesvirinae* of the *Herpesviridae* family (2). Previous reports indicated that pigs are the primary hosts of PRV (3, 4). Clinically, the manifestation of disease in pigs is related to the age of the pig, as young piglets usually present with severe central nervous system symptoms, and the outcome is invariably fatal. In contrast, elderly pigs usually present with mild respiratory signs or subclinical symptoms. Large-scale vaccination with *gE*-deleted vaccines may play an important role in controlling this disease. To date, PR has been eradicated in pigs in several European countries, the USA, and New Zealand (5–7).

Pseudorabies virus has a wide range of hosts, including pigs, cats, rabbits, cows, goats, cattle, sheep, dogs, bats, bears, coyotes, foxes, wolves, horses, deer, panthers, and some avian species (8). Unlike infections in pigs, fatal infections have been reported in several animals (including cats, dogs, and cattle) regardless of age. PRV is considered a zoonotic agent. In 2019, a PRV strain of human origin, hSD-1/2019, was isolated from a diseased human with acute encephalitis (9), providing solid evidence of the public health significance of PRV.

Cats have been confirmed as hosts for PRVs (10–12). Generally, cats are infected mainly through the ingestion of PRV-contaminated raw pork, particularly of the lungs and other offal. The virus generally enters through the oral route, replicates in the tonsil and pharynx, and spreads through the cranial nerve and the central nervous system. Cats infected with highly pathogenic PRV strains usually die within 48 h after the onset of the signs of this disease, which can include anorexia, pruritus, self-mutilation, ataxia, and, eventually, paralysis (13). The tolerance to PRV infection and trans-neuronal transport (12) in cats differs according to the neuronal cell types (13). However, the pathogenicity of attenuated PRV strains in cats remains unclear. In this study, we assessed the pathogenicity of attenuated PRV strains in cats and performed a retrospective analysis to monitor their epidemiological status. The results of our study will be beneficial for our understanding of the epidemiology and pathogenicity of PRV in cats.

## Materials and Methods

### Ethics Statement

This study was approved by the Animal Care Committee of the Institute of Animal Sciences of the Chinese Academy of Agricultural Sciences and animal experimental protocols (approval ID: IAS-2021-112). All study procedures and animal care activities were conducted in accordance with the recommendations of the Guide for the Care and Use of Laboratory Animals of the Ministry of Science and Technology of the People's Republic of China.

### Viruses and Animals

Attenuated PRV strain Bartha-K61 was purchased from YEBIO Co., Ltd. The pathogenic PRV strain SD18 (Batch No. MN443976), a variant PRV strain circulating in the Chinese swine industry, was isolated in the Shandong province of China in 2018 (14). In total, nine 10-week-old domestic cats were purchased from the Guangdong Research Center of Laboratory Animals (approval ID: SCXK-2013-0007). All animals were negative for PRV, as was detected by ELISA and PCR.

### Clinical Samples

From July 2019 to June 2020, a total of 54 feline samples (including nasal swab, heart, lung, liver, and brain) were collected in Guangdong province, China. Among these 54 feline specimens, 14 were collected from diseased cats that exhibited anorexia, pruritus, and ataxia; 9 were collected from diseased homeless cats around pig farms; and the remaining 31 were randomly collected from dead cats in pet hospitals. All samples were stored at −80°C.

### Virus Detection

The presence of PRV was detected using real-time quantitative PCR assay as previously described (14). Viral DNA was extracted from clinical samples using TRIzol reagent (Invitrogen). The specific primer pairs 5-TGAAGCGGTTCGTGATGG-3 and 5-CCCCGCACAAGTTCAAGG-3 targeting the PRV *gB* gene and the specific primer pairs 5-CCGCGGGCCGTGTTCTTTGT-3 and 5-GCGCCGGCGAGGTGAAGC-3 targeting the PRV *gE* gene were designed according to previous studies (14). Real-time quantitative PCR was performed as previously described (14). Briefly, the recipe contains 10 μl of a 2 × SYBR Premix ExTaq Green mix (TaKaRa), 1 μl of a template, and 0.5-mm concentration of specific primers. Thermal cycling parameters were as follows: 95°C for 5 min; 40 cycles of 95°C for 10 s, 58°C for 30 s, and 72°C for 30 s; and one cycle of 95°C for 30 s, 60°C for 30 s, and 95°C for 30 s.

### Challenge Study

To assess the virulence of different PRV strains in cats, nine 10-week-old cats were randomly divided into three groups (three cats per group). The cats in the first group were infected with the attenuated strain, Bartha-K61, at a dose of 10^3.0^ TCID_50_. The cats in the second group were infected with the highly pathogenic strain SD18 at a dose of 10^3.0^ TCID_50_. The cats in the third group were infected with phosphate-buffered saline as a negative control. Clinical signs of the disease were observed daily. All the cats were humanely euthanized until one of them presented with signs of disease or sudden death.

### Histopathology

Fresh pathological tissues were fixed in 10% neutral-buffered formalin, routinely processed, embedded in paraffin, sectioned (4-μm thick), and stained with hematoxylin and eosin according to standard protocols. Pathological changes were examined using light microscopy.

## Results

### The Prevalence of PRV in Cats

We collected 54 feline specimens from diseased or dead cats to perform a retrospective investigation. As a result, a total of five specimens were positive for PRV *gB* gene. Among these *gB*-positive samples, three were collected from diseased cats in pet hospitals, and two were collected from diseased homeless cats around pig farms (Table 1). Interestingly, all the PRV-positive samples from cats on pig farms were *gB*-positive and *gE*-negative. Among the PRV *gB*-positive samples from pet cats, one sample was negative for the PRV *gE* gene, whereas two samples were positive for PRV gE. These results showed that, of the five PRV-positive specimens, two were infected by wild virus strains, and three were infected by vaccine strains. These data provide clues to PRV infection in cats.

**Table 1:** Viral detection in a retrospective survey.

**Sample**	**Source**	**Tissues**	***gB***	***gE***
1	Pet cat	Nasal swab	+	+
2	Pet cat	Nasal swab	+	+
3	Pet cat	Nasal swab	+	–
4	Cat on pig farms	Nasal swab	+	–
5	Cat on pig farms	Lung, nasal swab	+	–

### PRV Cause Clinical Manifestation in Cats

**Figure 1:**
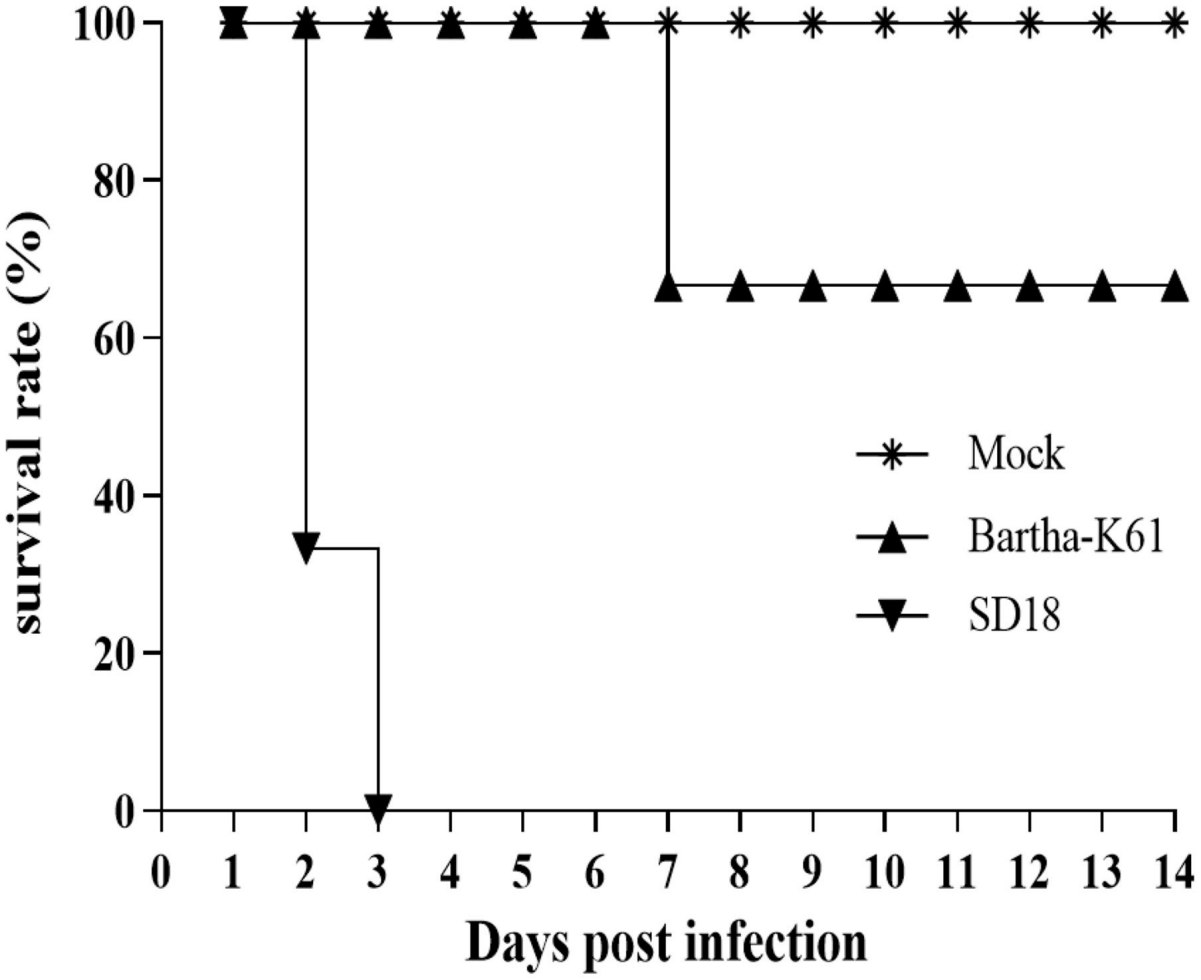
Survival curves of PRV-infected cats. Cats that were 10 weeks old were infected with Bartha-K61, SD18, or PBS. Clinical signs of disease were observed daily. In the Bartha-K61-infected group, one cat exhibited anorexia, anxiety, and crying and euthanized at the end of expeirence. Fourteen days later, the cats were euthanized with sodium nitrite.

PRV-infected cats usually present with anorexia and, sometimes, with intense pruritus, but few reports on the clinical signs caused by the attenuated PRV strain in commercial vaccines against cats are available. In this study, we infected 10-week-old domestic short hairs with the Bartha-K61 strain and recorded the clinical manifestations. The experiment was terminated 14 days post-infection (dpi). Compared to the cats in the mock-infected group, the death rate of the cats in the SD18-infected group reached 100%, while no deaths occurred in the Bartha-K61-infected group (Figure 1). Generally, all the cats in the SD18-infected group died or were euthanized at 2–3 dpi, following the presentation of nervous signs of disease. In the Bartha-K61-infected group, one cat exhibited anorexia, anxiety, and crying at 7 dpi, whereas the other two cats were euthanized at the end of the experiment. All mock-infected cats, without any signs of disease, were euthanized at 14 dpi.

Clinical observations were monitored during the experimental period. Compared to that of the mock-infected cats, the incubation period of SD18-infected cats was shorter: no longer than 2 days. All the cats presented with anorexia, pruritus, ataxia, and paralysis. The infected cats usually died within 24 h after the onset of clinical signs. In the Bartha-K61-infected group, the cats exhibited slight symptoms at 6–7 dpi, including anorexia, anxiety, and crying. Additionally, viruses were detected in nasopharyngeal swabs, anal swabs, and buccal swabs of the challenged cats at 1 or 2 dpi (Table 2).

**Table 2:** Virus detection in the excretion of challenged cats.

**Group**	**Swab samples**	**Days post-infection**							
		**0**	**1**	**2**	**3**	**4**	**5**	**6**	**7**
Mock	Buccal	0/3	0/3	0/3	0/3	0/3	0/3	0/3	0/3
	Nasopharyngeal	0/3	0/3	0/3	0/3	0/3	0/3	0/3	0/3
	Anal	0/3	0/3	0/3	0/3	0/3	0/3	0/3	0/3
Bartha-K61	Buccal	0/3	0/3	2/3	2/3	3/3	3/3	3/3	3/3
	Nasopharyngeal	0/3	0/3	2/3	3/3	3/3	3/3	3/3	3/3
	Anal	0/3	0/3	2/3	3/3	3/3	3/3	3/3	3/3
SD-18	Buccal	0/3	2/3	3/3	/	/	/	/	/
	Nasopharyngeal	0/3	2/3	3/3	/	/	/	/	/
	Anal	0/3	1/3	3/3	/	/	/	/	/

### PRV Cause Pathological Lesions in Cats

**Figure 2:**
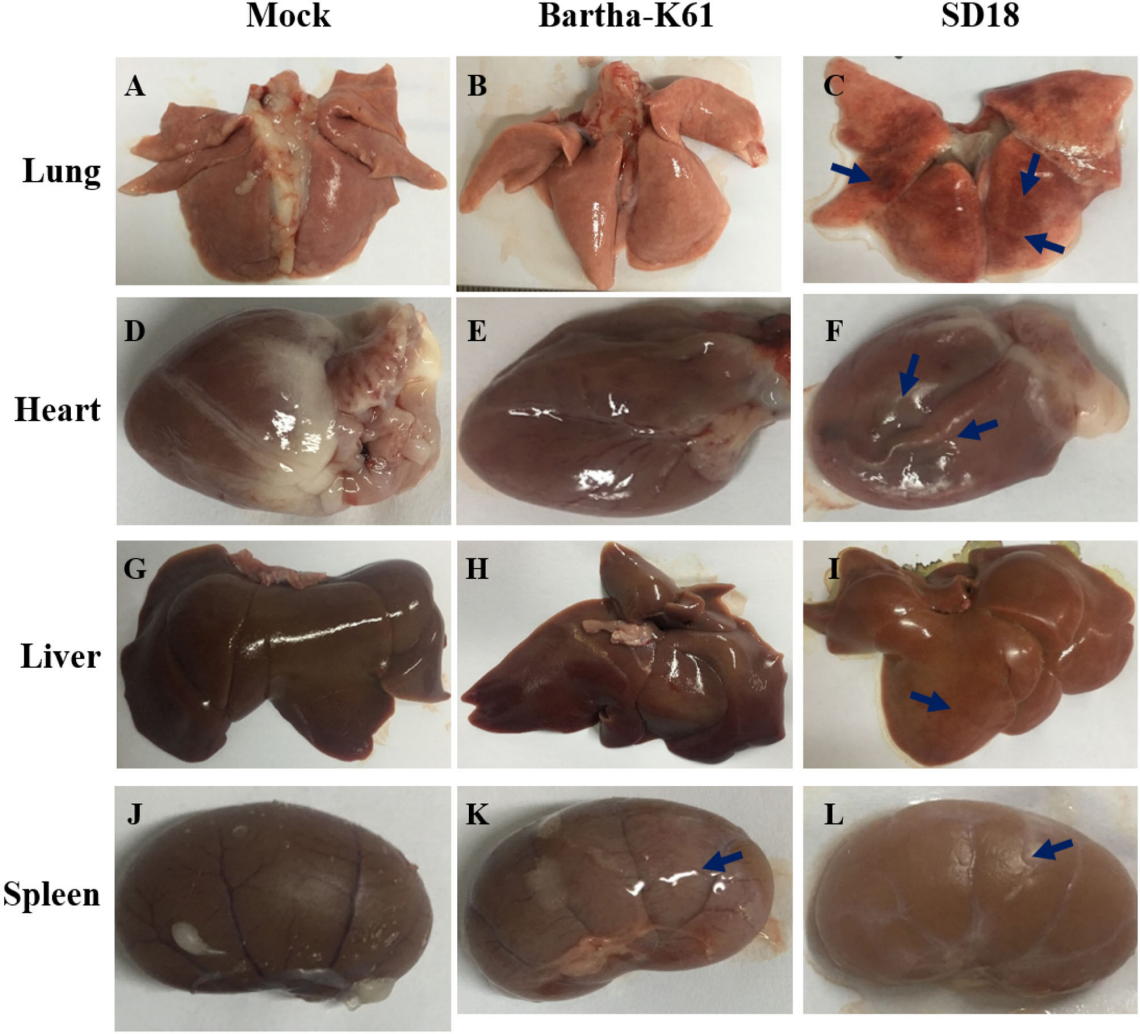
Pathologic changes in PRV-infected cats. Cats that were 10 weeks old were infected with Bartha-K61, SD18, or PBS. All the cats were euthanized at 14 dpi. The autopsy was performed, and the gross abnormalities were recorded at necropsy. **(A–C)** The necropsy symptoms of the lung. **(D–F)** The necropsy symptoms of the heart. **(G–I)** The necropsy symptoms of the liver. **(J–L)** The necropsy symptoms of the kidney.

To further assess the pathogenicity of the attenuated PRV strain in cats, we performed an autopsy and recorded gross abnormalities at necropsy. Compared to the mock-infected cats, the SD18-infected cats presented with apparent lesions, while the Bartha-K61-infected cats exhibited slight symptoms of disease at necropsy (Figure 2). Generally, SD18-infected cats presented with severe hemorrhages and congestion in the lung (Figure 2C), swelling in the kidney and heart (Figures 2F,L), and focal hemorrhage in the liver (Figure 2I), which is consistent with previous reports (11). Exceeding our expectations, the Bartha-K61-infected cats did not show typical apparent lesions, except for kidney swelling (Figure 2K).

### Histopathological Analysis of PRV in Cats

**Figure 3:**
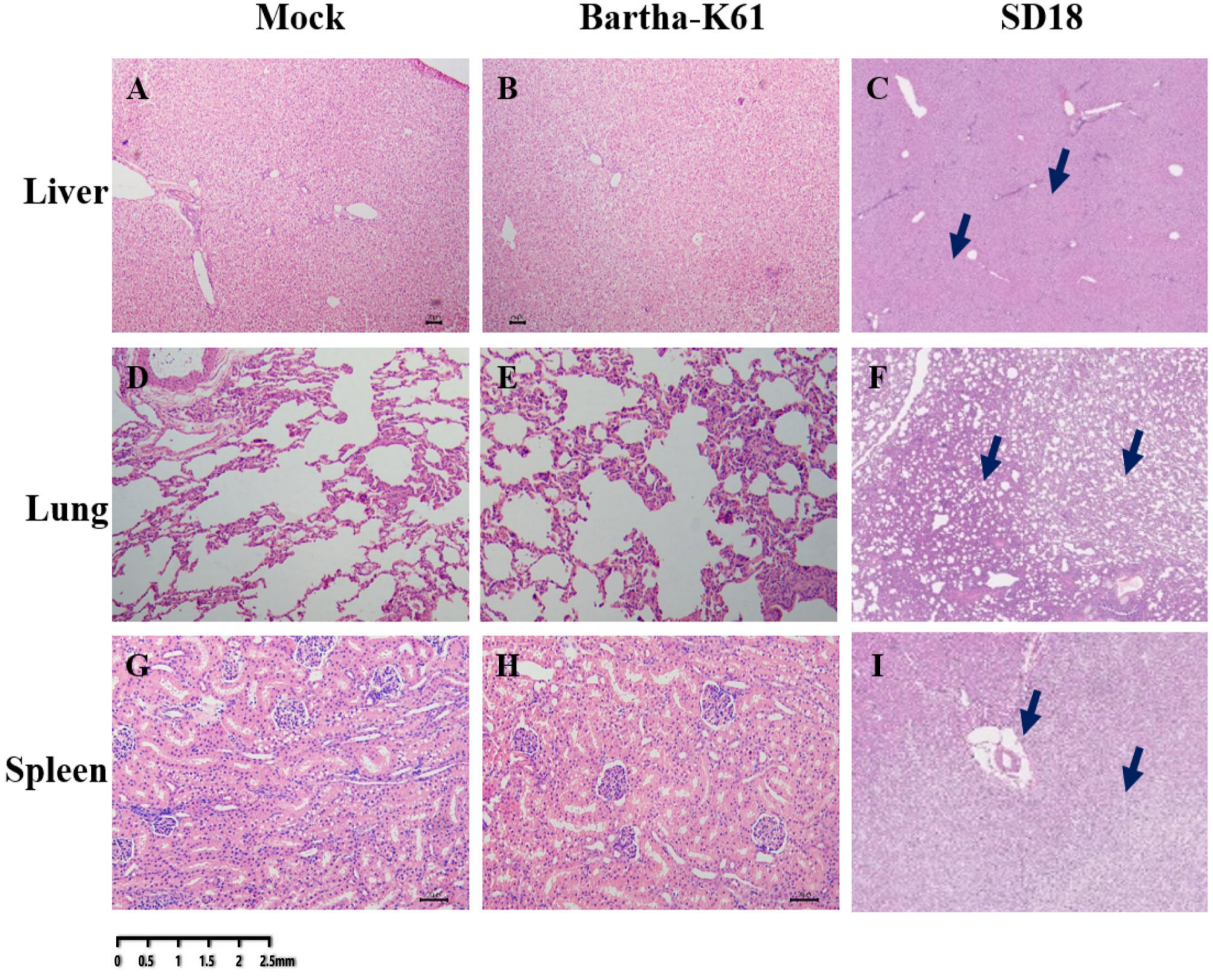
Histological changes in PRV-infected cats. Affected tissues collected from the mock-, BarthaK61, and SD18-infected cats were used for histological analysis. Histological changes were examined by light microscopy. **(A–C)** Histological changes of the liver. **(D–F)** Histological changes of the lung. **(G–I)** Histological changes of the spleen.

To further analyze the pathogenicity of the attenuated PRV strain in cats, histopathological analysis was performed. As a result, compared to the sham-infected cats, the BarthaK61-infected cats only presented with lesions in the kidney (Figure 3), which is consistent with the observations at necropsy. However, SD18-infected cats presented with severe multiorgan lesions. Generally, cellular degeneration, as was observed in the liver and kidney of the SD18-infected cats (Figures 3A–C,G–I), severe hemorrhage, and congestion were observed in the lungs of the SD18-infected cats (Figures 3D–F).

## Discussion

PRV was first identified in a cat in China and subsequently isolated from many species of animals (15), including pigs, cattle, cows, goats, dogs, rabbits, some avian species, and humans (16). Pigs are considered the primary hosts and reservoirs of PRVs (3, 4). Interestingly, PRV strains circulating in many animal species may be transmitted from swine because of the high homology of genomic sequences between PRV isolates from these animals and the commercial vaccine strains or swine epidemic strains (14, 17, 18). Our previous studies confirmed the transmission route of PRV from pigs to dogs (14, 18). Cats are usually infected with the PRV through direct contact with PRV-contaminated raw pork (19). Of course, the route of infection may be indirect. Viruses can be transmitted *via* viral excretion or by virally contaminated fomites. A series of case reports on the outbreak of this disease in sheep, cats, and dogs have confirmed this transmission route (20). More efforts are required to monitor viral epidemics and morbidity in cats.

With the presence of companion animals, cats have become increasingly ubiquitous in human life, resulting in a hidden threat to humans due to the susceptibility of cats and humans to PRV. Although the occurrence of this disease in cats is sporadic, PR disease should receive more attention because it is easily mistaken for rabies, and death occurs rapidly. Thus, it is necessary to investigate the prevalence of PRV in cats, especially pets. Previous reports have indicated that cats can be infected with highly pathogenic PRV mainly through contact with PRV-contaminated raw pork and die within 12–48 h after the onset of clinical signs (13). Considering the widespread use of attenuated PRV strains in commercial vaccines, we speculated that PRV might be widely distributed in cats. To test this hypothesis, we performed a retrospective investigation of PRV infection in cats. Our data provide evidence that *gE*-deleted PRV strains are prevalent in Chinese cats. Unfortunately, no virus was successfully isolated, owing to low viral titers in swab specimens and the long storage time of the samples. More efforts are required to monitor PRV-infected cats for virus identification.

The pathogenicity of highly pathogenic PRV strains in cats was assessed. According to previous publications, the incubation time of PRV in cats is ~48–72 h, and death mainly occurred within 12–48 h after the onset of typical symptoms of this disease. To date, the pathogenicity of attenuated PRV strains in cats has remained unclear. Therefore, in this study, we infected cats with an attenuated PRV strain to evaluate its pathogenesis. We used the attenuated PRV strain Bartha-K61 as the viral template because Bartha-K61 is widely used in the Chinese swine industry, resulting in relatively favorable control of this disease (16, 21, 22). Similar to previous publications (11), the incubation period of highly virulent PRV infection in cats was ~24–48 h, and all the cats died within 24 h after the onset of clinical signs. In contrast, the Bartha-K61-infected cats did not present any neurological symptoms, except for anorexia, anxiety, and crying. To confirm the association of signs with Bartha-K61, we performed an autopsy of a Bartha-K61-infected cat and recorded gross abnormalities at necropsy. Interestingly, no pathological changes were observed. It appears that Bartha-K61 is not pathogenic to cats. However, why do Bartha-K61-infected cats present clinical signs? The impaired intra-axonal transport process may affect replication, the spread, and tissue tropism of the Bartha strain *in vivo*. Further effort is required to study the involvement of Bartha-K61 with clinical signs in cats.

## Data Availability Statement

The original contributions presented in the study are included in the article/supplementary material, further inquiries can be directed to the corresponding author.

## Ethics Statement

The animal study was reviewed and approved by the Animal Care Committee at the Institute of Animal Sciences of the Chinese Academy of Agricultural Sciences approved the animal experiment protocols (approval ID: IAS-2021-112).

## Author Contributions

RL and LT designed the study, interpreted the data, and gave final approval of the version to be published. LT, JZ, QC, SZ, LL, XT, and SH performed the experiments. WY mainly responsible for the collection of samples. All authors contributed to the article and approved the submitted version.

## Funding

This work was supported by the Central Public-Interest Scientific Institution Basal Research Fund (Grant Nos. 2020-YWF-YTS-10 and 2022-YWF-ZYSQ-12) and the Agricultural Science and Technology Innovation Program of China (ASTIP-IAS15).

## Conflict of Interest

The authors declare that the research was conducted in the absence of any commercial or financial relationships that could be construed as a potential conflict of interest.

## Publisher's Note

All claims expressed in this article are solely those of the authors and do not necessarily represent those of their affiliated organizations, or those of the publisher, the editors and the reviewers. Any product that may be evaluated in this article, or claim that may be made by its manufacturer, is not guaranteed or endorsed by the publisher.
